# Chemometrics and Standards

**DOI:** 10.6028/jres.093.017

**Published:** 1988-06-01

**Authors:** L. A. Currie

**Affiliations:** Center for Analytical Chemistry, National Bureau of Standards, Gaithersburg, MD 20899

## 1. Introduction

*Standards* are central to the achievement and maintenance of accuracy in trace analysis. This fact is well-known and well-accepted in the international analytical chemical community, where “standards” are generally considered to be *Standard Reference Materials* (SRMs) or *Certified Reference Materials* (CRMs). The term, standards, however, is multivalued, as noted recently by a former Director of the National Bureau of Standards [1]. That is, even in our more conventional view of trace analysis, we must consider in addition to standard materials: standard procedures (protocols), standard data (reference data), standard units (SI), standard nomenclature, standard (certified) instruments, and standard tolerances (regulatory standards, specifications, norms) [2]. It is interesting, in light of these several types of “standards” which have some bearing on accuracy in trace analysis, to consider the possible significance of standards in and for Chemometrics.

To pursue this objective, we first must have a common understanding of the meaning of the term, chemometrics, and what significance it may have for accurate trace analysis. A concise definition is given by the subtitle of the volume which resulted from the first NATO Advanced Study Institute on Chemometrics, i.e., “Mathematics and Statistics in Chemistry” [3]. Implications for accuracy, especially accuracy in trace analysis, are immediately evident That is, wherever mathematical or statistical operations contribute to the experimental design, data evaluation, assumption testing, or quality control for *accurate* chemical analysis, “chemometric standards” are at least implicitly relevant.

The major part of this paper will be devoted to an explicit discussion of such chemometric standards, including case studies drawn from recent research at the National Bureau of Standards. The discussion will be placed in the framework of the Analytical System, or Chemical Measurement Process (CMP), for such a perspective makes it possible to consider logically a “theory of analytical chemistry”; and certainly chemometrics is a very important part of such a theory [4,5]. To set the stage, the next section will include a brief view of the current content of Chemometrics, together with a summary of its history and literature. This article will conclude with a glimpse at the future of chemometrics, with special emphasis on means to achieve increased accuracy in our chemical measurements and increased understanding of the external (physical, biological, geochemical) systems which provide the driving forces for analytical chemistry.

## 2. A Brief History

The content of Chemometrics, as viewed by the “Working Party on Chemometrics” of the Union of Pure and Applied Chemistry (IUPAC), is given in table 1 [6]. Included in the second, major portion of the table are titles for some 30 chapters which comprise an overview document being prepared for IUPAC. Two points are evident from the list of titles: (1) the scope of chemometrics is very broad indeed, encompassing significant portions of applied mathematics; (2) as implied by the name, major emphasis is given to measurement, specifically chemical measurement. In a narrower sense, chemometrics is sometimes viewed as the intersection of statistics and analytical chemistry, as seen by the emphasis on experimental design, control, and the analysis of signals and analytical data. The several chapters on signal and data analysis include such topics as filtering, deconvolution, time series analysis, exploratory data analysis, clustering, pattern recognition, factor analysis, and (multivariate) regression. Standards and analytical accuracy have special relevance to the chapters on terminology, precision and accuracy, performance characteristics, calibration, analysis, and quality control.

A brief, chronological history of chemometrics is presented in table 2. To convey information on both the history and the literature of this discipline, we have indicated milestones in the form of selected references, to the extent possible. Impressive, recent growth is seen by the fact that the first two textbooks and the first two journals, specifically devoted to chemometrics, were published within the last 2 years. Looking to the beginning of this history (bottom of table 2), we find the name of Jack Youden, certainly one of the earliest and most notable chemometricians, whose excellent guide to chemometrics was published some 20 years prior to the invention of the term. (Youden, incidentally, was a *proper* chemometrician, in that he began his career as a chemist, and then went on to become a distinguished statistician.) The journal *Analytical Chemistry* has long served chemometrics well, through its biennial fundamental reviews of the subject, starting well before the term was known. As indicated in table 2, the term “chemometrics” was conceived by Svante Wold, in January 1971. The reader’s attention is called to the interesting paragraph by Wold, in reference [7], which details the beginnings of chemometrics, including the start of the Chemometrics Society by Wold and Kowalski, in Seattle on 10 June 1974. The intervening decade, culminating in the forementioned NATO Advanced Study Institute, saw rapid growth in chemometrics education and research, much of it promulgated by the Chemometrics Society and published in journals such as *Analytical Chemistry* and *Analytical Chimica Acta*. Also, there appeared several notable texts which were largely chemometric in content, if not in title [8–13].

To complete this brief look at the content, history and literature of chemometrics, it is fitting to refer to the Chemometrics Conference held at NBS just 3 years ago. It was a special meeting in many respects, for it epitomized the interdisciplinary nature and increasing scope of chemometrics; and it was “probably the first (such meeting) in the United States by that title” [14]. The meeting was jointly planned by an interdisciplinary team, consisting of a chemist and two statisticians. It was jointly sponsored by two national chemical and two national mathematical societies. Finally, it contained an extremely effective and balanced blend of experts from the two disciplines: mathematicians (and statisticians) providing critiques of chemometrics presentations by chemists, and chemists providing critiques of the presentations by mathematicians. The synergism resulting from this approach is evident from examining the proceedings [14]. It is appropriate to conclude with reference to this volume, for it was dedicated to W. J. Youden, our first chemometrician in table 2.

## 3. Chemometric Standards and the Analytical System

### 3.1 Standards

The agenda for chemometrics, from the perspective of standards, is outlined in table 3. First, we must deal with the issue of nomenclature. Because of the relatively recent formal emergence of chemometrics, and because of its interdisciplinary character, this is a very important matter for our early attention. Nomenclature, in this context, refers to much more than terminology. That is, it includes basic meaning and explicit formulation of concepts falling within the scope of mathematics and chemistry. The efforts of IUPAC, both in the Commission on Analytical Nomenclature [15] and as outlined in table 1 [6], will be extremely helpful in this fundamental task for chemometrics—to assure that chemists and mathematicians “speak the same language” where that language maintains as much self consistency as possible with the slightly diverse languages of the separate disciplines. (To some extent, we shall have to accept a bilingual dictionary. For example, “efficiency,” “consistency,” and “sample,” have somewhat different implications in statistics and analytical chemistry.)

Supporting standards for accuracy, for the entire Chemical Measurement Process, is perhaps our most important task. The primary components are indicated under the second heading in table 3. Most important is a rigorous approach to the specification and evaluation of the fundamental characteristics of analytical methods and analytical results, such as detection, identification, and quantification (estimates and uncertainties). A combination of chemical knowledge (or “chemical intuition”) and statistical expertise in this effort is the best means to assure validity and control through the specification and testing of assumptions. A second level of control which represents a special responsibility for chemometrics is the production and evaluation of quality software and algorithms—a responsibility which is being met in both chemometrics journals. The logical extension of chemical software standards is found in chemometric validation, or Standard Test Data (STD), designed to guarantee quality for the Evaluation step of the CMP. STD thus parallel SRMs for accuracy assurance in both intra- and interlaboratory environments [16]. It is worth emphasizing that with the enormous progress in laboratory automation, and the substitution of machine intelligence for human intelligence, quality control of the mathematical or chemometric phase of the CMP becomes ever more urgent. Direct instrument responses are increasingly unavailable for the expert scrutiny of the analyst, and automatic results are produced with little indication of the assumptions involved or numerical validity (and robustness to outliers) of the computational methods.

The last “standard” indicated in table 3 relates to design. Design of the sampling, measurement, and data evaluation steps of the CMP to meet specified needs, is really the *first* responsibility of chemometrics. A careful blend of statistical expertise and chemical knowledge once again is the best means for meeting the accuracy or information requirements of the CMP. Inadequate attention to design is perhaps the most serious fault in ordinary chemical analysis. Either inconclusive or inadequate chemical results are obtained, using the samples and methods at hand, or costs are needlessly high in obtaining the relevant information. This area constitutes one of the greatest opportunities for chemometrics for attaining requisite accuracy at minimal cost; appropriate methods include information and decision theory, statistical design and optimization techniques, and exploratory multivariate approaches such as pattern recognition and cluster analysis [3].

### 3.2 The Analytical System

A “systems perspective” for the CMP has been promulgated by a number of eminent analytical chemists over the past 2 decades. One of the earliest and most noteworthy efforts was made by the *Arbeitskreis “Automation in der Analyse”* beginning in the early 70s [4]. The systems and information theoretic view, which was pioneered by members of this circle, such as Gottschalk, Kaiser, and Malissa, is even more relevant today, and it offers perhaps the best model for an integrated chemometric approach to accuracy. Considering a simplified representation of the CMP or analytical system presented for this purpose in reference [16] (fig. 2), for example, it is clear that not only is there material flow through the system, in terms of sampling, sample preparation, and measurement, but there is also the flow of information, and unfortunately noise. Treating the CMP as an integrated system is essential for the optimal application (cost vs accuracy) of chemometric tools for design, control, calibration, and evaluation. Interfaces between the several steps of the CMP must be astutely matched to prevent information loss, and data evaluation and reporting techniques must be recognized as part of the overall measurement process, capable of preserving or distorting information just like the chemical and instrumental steps. The CMP or analytical system model can be especially helpful in planning for accuracy through appropriate points of introduction of SRMs and STD, and for explicit treatment of feedback, where initial results are utilized for improved, on-line redesign (“learning”) of the CMP.

Extended discussion of the analytical system is beyond the scope of this paper, but its introduction is essential for a meaningful consideration of the relationship of chemometrics to accuracy and standards, as indicated above. The system view is obviously important for designing or investigating overall performance characteristics, such as the blank, recovery, specificity, and systematic and random error—through propagation techniques [17]. That is, if one wishes to achieve an overall precision, or detection limit, or identification capability, then the design of an optimal system must take into consideration the corresponding parameters for each step of the CMP, from sampling through data evaluation. *Such an integrated approach to design, with the help of chemometric techniques, is as relevant to the design of self-contained automated and intelligent analytical instruments, as it is to the design of an integrated analytical approach of an entire organization (such as *CAC*) to a broader analytical question, such as the selection and certification of Standard Reference Materials* [4, 12].

### 3.3 Hypothesis Testing and the CMP

Fundamental questions to be addressed by measurement science can often be posed as hypotheses, to be tested or evaluated via analytical measurements. The formation of meaningful hypotheses or models of the external (environmental, biological) system is the business of expert scientists within that discipline. The testing of such hypotheses, through analytical measurements, is the business of expert analytical chemists. From this perspective it is clear that hypothesis testing captures the essence of the scientific method. It *must* therefore be a key feature of any “theory of Analytical Chemistry.” This is especially important for chemometrics, for hypothesis testing forms one of the cornerstones of modern statistics. By capitalizing on the elegant statistical tools that have been developed for agricultural or biological testing, for example, we can generate an objective and optimal approach to the design of the CMP. That is, by combining excellent knowledge of chemistry with that of modern statistics, we can construct CMPs which are guaranteed to have sufficient (statistical) power to meet the specified analytical needs. In this respect, we shall be responding to a famous challenge by Kaiser [18], that analytical chemists learn to match optimally the “space of analytical methods” with the “space of analytical problems.”

**Figure 1:**
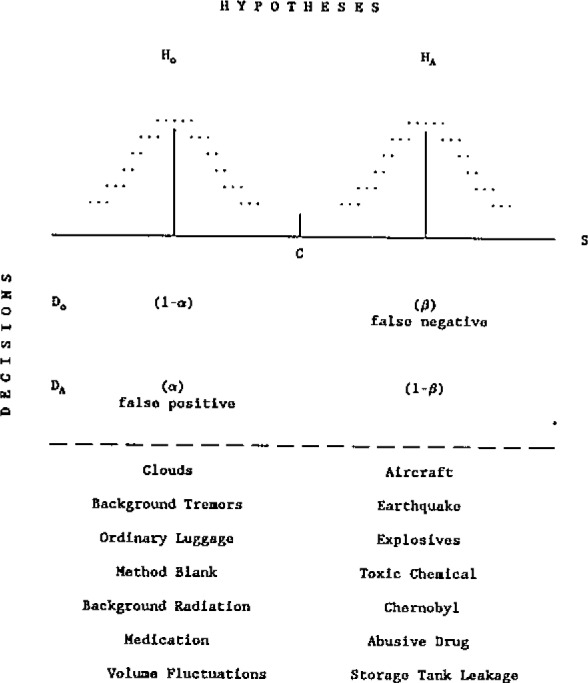
Hypothesis testing and societally important detection decisions. Sets of null (H_o_) and alternate (H_A_) hypotheses are listed below a Truth Table and stylized probability density functions [19].

**Figure 2:**
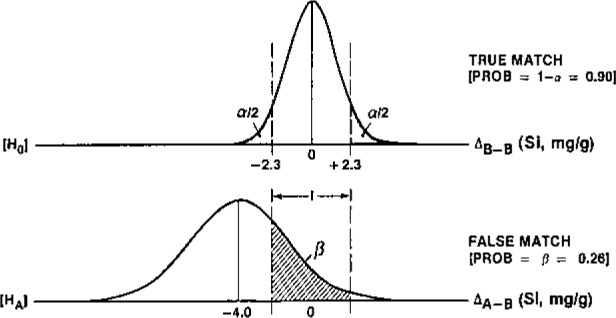
Hypothesis testing formulation for identification in analytical chemistry. Probability density functions are given for the difference in composition (*Si*) for particles emanating from the same source vs two different sources [19].

**Figure 3:**
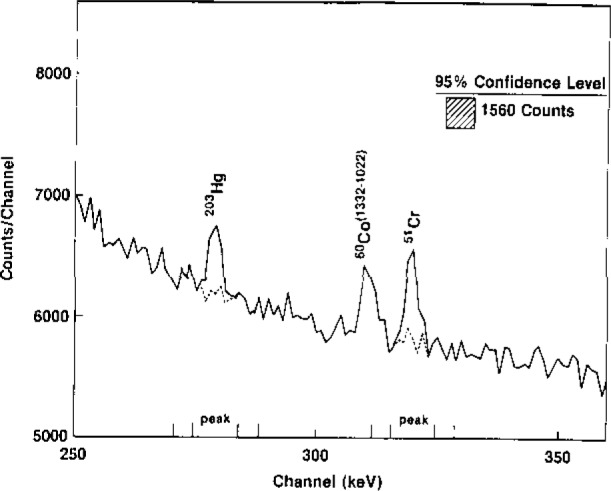
Clearly visible gamma ray peaks (^203^Hg, ^51^Cr), which were not detected above a ^60^Co background in the IAEA practical examination of commercial software [20].

Some of the ways in which hypothesis testing impacts analytical accuracy, in terms of the fundamental parameters of analytical chemistry, are presented in table 4. Figures 1 and 2 convey the elements of this theory together with its application to detection and univariate identification, respectively [19]. Further details cannot be presented here, but it should be noted that accuracy in trace analysis demands quantitative chemometric approaches to detection, identification, and quantification (uncertainty evaluation), plus model and assumption validation. Inadequate attention to this matter, and imperfect understanding of the fundamental (*α, β* errors) limitations of hypothesis testing, i.e., chemical measurement, continue to produce very erroneous conclusions regarding the results or power of our analytical techniques [19]. It is especially interesting and important to consider this in terms of the final, data evaluation step of the CMP, in view of the expanding use of “intelligent” and automated instrumentation, which generally includes “black box” data evaluation. Monitoring the accuracy of such internal algorithms is clearly one of the critical tasks of chemometrics in the near future, one for which Standard Test Data (STD) may play an important role. The need is exhibited in figure 3, where perfectly visible gamma ray peaks remain “undetected” by a widely used instrumental gamma ray analytical system [20].

Before leaving this survey of fundamentals, we must emphasize the importance of the first syllable. Chemometrics differs from statistics and mathematics in that chemical intuition or expertise forms an essential part of the activity. As mentioned above, hypothesis formation, which is necessarily the first step in designing a scientific experiment, requires disciplinary expertise. Accuracy in data evaluation or experiment control, for example, can only be expected when the chemometric techniques employed recognize the range of possible alternative hypotheses (models or assumptions). This is the crux of setting reliable bounds for systematic error, or in establishing “definitive” analytical methods. Empirical rules or heuristic techniques adapted to this purpose should be viewed with some caution. Examples of problems demanding chemical expertise for alternative hypotheses are identification, and the assessment of blank and matrix effects [17, 19 (ch. 16)]. In figure 2, for example, knowledge of the alternative was essential to compute the identification power of the test. In the more general case, where chemical species are identified on the basis of spectral or chromatographic patterns, we must know the locations and uncertainty characteristics of *all* “nearby” patterns to assess the identification power for a given null pattern, or to design a measurement process meeting prescribed identification capabilities. In moving from the universe of all possible neighboring spectral patterns, to the universe of possible interferences [21] or calibration models, for example, chemometrics faces a considerable challenge.

## 4. Selected Illustrations

To illustrate the relevance of chemometrics to the assurance of accuracy in trace analysis, we shall examine three recent and continuing investigations from our laboratory. The first has been selected as an example where quantitative hypothesis testing techniques have been applied to one of the fundamental elements of any analytical system: the noise. The second relates to an exploratory research study which seeks to relate patterns of laser microprobe mass spectra to sources of combustion particles (“soot”) in the atmosphere. It illustrates the importance of *chemical* information (or “intuition”) to maintain accuracy in the application of multivariate data analytical techniques. The third illustration speaks to the need for STD, both for monitoring accuracy in complex chemical data evaluation, and as a stimulus for research for improved chemometric techniques and understanding of the data evaluation process.

### 4.1 Counting Statistics—Are They Poisson?

The two fundamental model characteristics of analytical signals are the functional relation, connecting the expected value of the signal to the analyte concentration, and the error structure, as indicated in table 4. Accurate measurements and accurate assessment of method performance characteristics demand knowledge of both. In this section we describe an experiment designed to investigate the statistical properties and the causal characteristics of the noise component in counting experiments. Such experiments, where individual atoms, ions, or photons are counted, comprise some of the most sensitive in analytical measurement. In many such cases it is assumed that the limiting counting noise is Poisson in nature. Since the variance of the Poisson distribution is equal to the mean, such an assumption leads to a simple error (standard deviation) estimate, and error propagation techniques may then be used for estimating uncertainties for net signals and analyte concentrations.

The primary objective of our investigation of noise was to test the validity of the Poisson hypothesis for very low-level counting data, with special emphasis on background counts. The validity of the Poisson assumption has long been one of the more intriguing questions in nuclear physics and chemistry, and it has therefore been the subject of some notable experiments [22]. Our experimental system was uniquely designed to permit a much more stringent test of this hypothesis, as it provided individual arrival times for more than a million events. A second objective, if the Poisson assumption proves valid, is to provide a physical random number generator—a device operating by the laws of physics, to generate random numbers for use in numerical simulations, as an alternative to numerical pseudo-random numbers.

**Figure 4:**
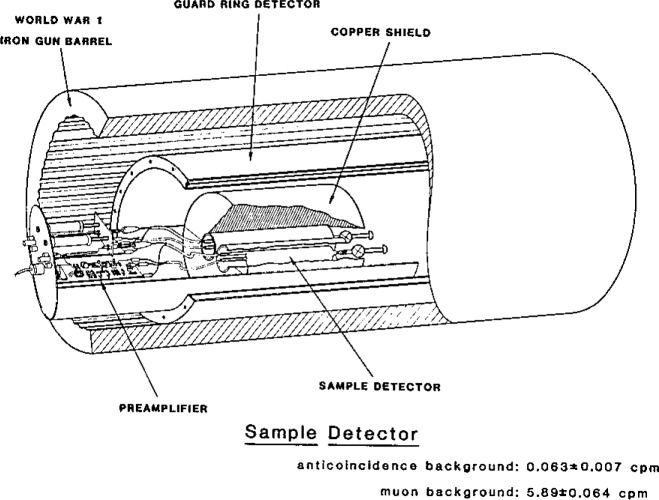
Low-level counting system. Penetrating cosmic rays (mu mesons) are removed as a background component of the sample detector by coincidence with the guard ring detector, reducing background by two orders of magnitude. (Uncertainties shown correspond to the Poisson standard deviations for a 24 hour counting period.)

**Figure 5:**
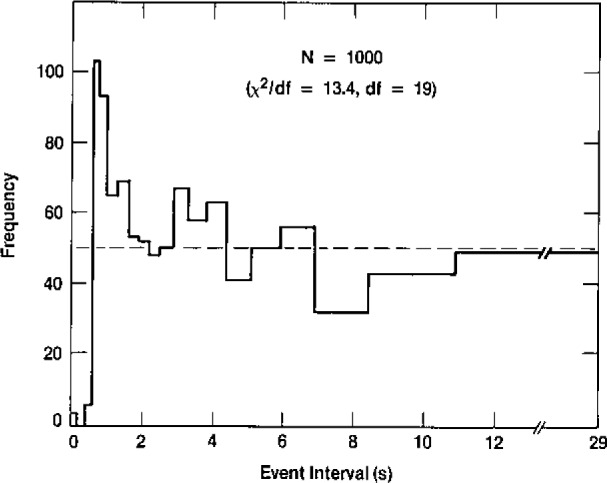
Chi-square test of the empirical equal probability histogram for low-level counting data [23].

A practical objective for investigating the low-level counting noise distribution derives from our physical knowledge of the measurement system, i.e., our knowledge of potential alternative hypotheses. Perhaps the most important such alternative is the possibility of correlated events in the radiation detector, which could have a profound influence on the magnitude and variability of our background noise. As indicated in figure 4, the effective background is reduced by about a factor of 100 through anticoincidence shielding. If, due to wall or gas impurity effects, just 1% of the electronically canceled events were to produce a secondary, time delayed event in the central detector, the effective background would be doubled! Time series and distributional analysis of the background noise thus allows us to investigate this alternative process. Knowledge of the statistical power of the null (Poisson) hypothesis test against this particular alternative is therefore vital both for the construction of valid uncertainty intervals, and for understanding the basic physics and chemistry of the background events. One illustration of the distributional analysis is given in figure 5, where χ^2^ is used to test deviations from the expected exponential distribution of time intervals between events. Further discussion of this investigation, including a tabulation of six alternative hypotheses, is given in reference [23]. Further investigation of sources of background noise is currently underway, using multivariate exploration of pulse shape characteristics.

### 4.2 Multivariate Exploratory Analysis: Origins of Atmospheric Soot Particles

Perhaps the best known applications of chemometrics involve multivariate techniques such as principal component analysis (PCA) and cluster analysis. Such techniques have reached a high degree of sophistication, as exploratory tools for the classification of samples which may be characterized by multivariable patterns or “spectra.” An excellent introduction to the principles and methods of the “soft” or empirical multivariate modeling techniques is given in reference [24]. PCA and related techniques are especially useful for data exploration, in that they permit ready visualization of sample relationships, provided there are not too many independent components in the system under investigation. Thus, a collection of mixtures of two components having quite complex, yet different, spectra or chemical patterns, can be represented by a set of points in a plane, or on a line if the mixtures are normalized. If the pure components are represented, they appear as the end points. Two dimensional PCA plots thus allow us to display relations among mixtures of three normalized components; and three dimensions increases the display capability to four components. Beyond exploratory display capability, several methods of multivariate chemical analysis may be employed for quantitative estimates for the number and identity of components, and for the analysis of mixtures [25]. These are outgrowths of the seminal work of Lawton and Sylvestre [26].

**Figure 6:**
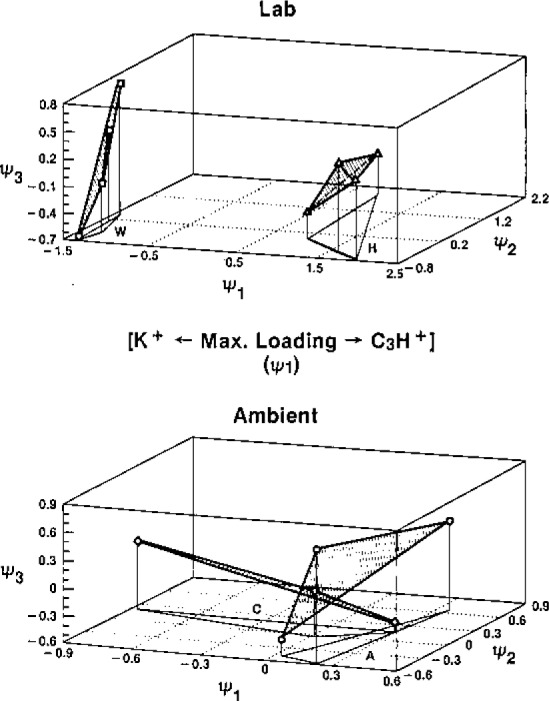
Isometric PCA projections of Lab and Ambient particle LAMMS positive ion spectra on the first three eigenvectors. Soot particles from wood are denoted “W” and “C”; those from hydrocarbon fuel are denoted “H” and “A.” Feature (mass) selection on the basis of “characteristicity” preceded the principal component analysis [28].

**Figure 7:**
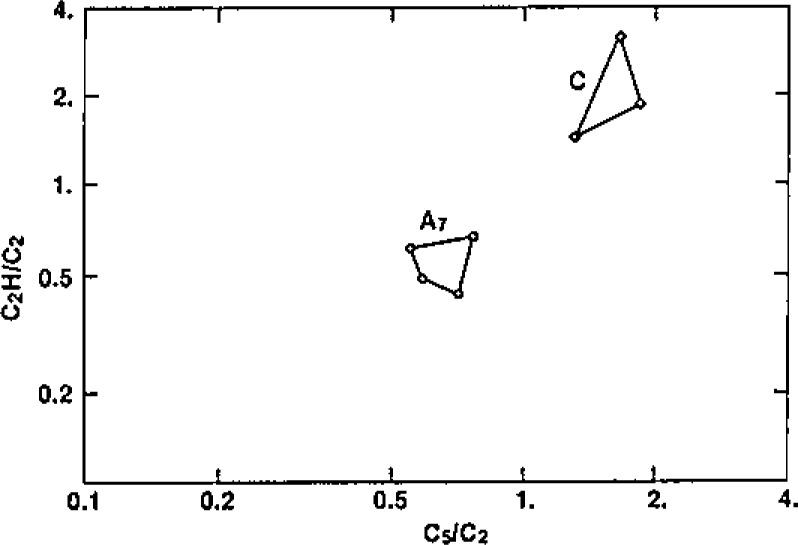
Bi-plot showing negative ion carbon cluster discrimination of LAMMS spectra from ambient atmospheric soot formed from the combustion of hydrocarbon fuel [“A”] and wood [“C”].

The interplay between the multivariate display techniques and chemical “intuition” (experience, knowledge) is exhibited in our investigation of laser microprobe mass spectra (LAMMS) of individual soot particles formed from the combustion of wood and fossil fuel. The scientific basis for our interest in this problem derives from the potential health effects of combustion particles, which often carry mutagens, on the one hand, and the geochemical and climatic implications, on the other. The ability to infer combustion sources for individual soot particles could add greatly to our understanding of climatic perturbations and perhaps even such phenomena as the Tertiary-Cretaceous Extinction [27]. PCA data exploration was attractive for this study because the system was relatively simple in terms of intrinsic structure (two components), but relatively complex in terms of both the graphitic soot formation and laser plume ion formation processes. The work demonstrates an extremely important point with respect to accuracy, however. That is, the importance of having thoroughly reliable chemical information for validation of the exploratory techniqres. This is shown in figure 6. The upper part of the figure shows the successful classification of wood vs hydrocarbon fuel soot particles on the basis of their positive ion laser microprobe mass spectra. Application of this model, which was developed for laboratory-generated particles, to soot particles collected in the field (urban atmosphere), however, would lead to erroneous conclusions (misclassification). The failed classification shown in the lower part of the figure was discovered through the use of an independent tracer of *known accuracy*, ^14^C, for source discrimination [28]. Subsequent research on this very important basic and practical problem has led to some understanding of the reason for the difference between laboratory and field particles, a basic issue being sensitivity of certain species (features) to deviations from the two-source, linear model. This example illustrates one of the more important cautions in the use of multivariate techniques, such as PCA and factor (FA) analysis: namely, the influential character of outliers and departures from assumptions. Further investigation of the atmospheric particles has shown the utility and relative robustness of selected negative ion carbon clusters for combustion source discrimination, as shown in figure 7. Unlike PCA and FA approaches to exploratory multivariate data analysis, the coordinates of the “bi-plot” of figure 7 are *not perturbed by outliers*. Also, they are often more readily interpretable chemically than eigenvectors, though clearly they do not possess the dimension reduction efficiency of PCA.

### 4.3 Standard Test Data

A special task for chemometrics is guaranteeing the accuracy of the data evaluation phase of the chemical measurement process. An important element in the task is the development of representative, reference data sets having known characteristics, for testing the validity of data evaluation. Such “standard test data” (STD) thus play the same role for data evaluation that SRMs do for procedure evaluation. STD are likely to become increasingly important as the data evaluation step becomes more complex, and as it becomes less accessible to the user, as in automated analytical systems. The nature and importance of STD for assessing interlaboratory precision and accuracy have been well demonstrated by exercises based on univariate gamma ray spectral data created by the International Atomic Emergy Agency (IAEA) [29] and multivariate atmospheric data created by NBS [30]. The parallelism with SRMs has been further established for the former STD through incorporation into the catalog of the IAEA’s Analytical Quality Control Service Program [31]. A brief description of the objectives and outcome of the multivariate STD exercise follows. (A more extended review of both exercises may be found in reference [16].)

**Figure 8:**
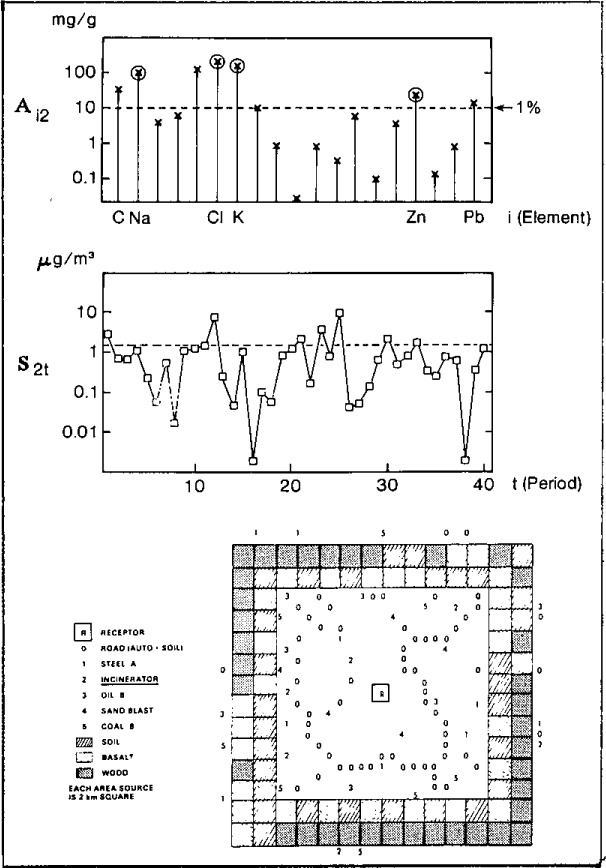
Source apportionment STD. A_i2_ represents the source profile vector for source-2 (incinerator); S_2t_ represents the source intensity time series for the same source. The lower portion of the figure shows the aerosol source emission map [16].

The objective of the multivariate STD exercise was to evaluate the resolving power, and precision and accuracy of all major mathematical techniques employed for aerosol source apportionment, based on linear models incorporating chemical “fingerprints” or spectra. To adequately test these techniques, which comprised various forms of multivariate factor or regression analysis, it was necessary to generate data matrices which were realistic simulations of the variations in source mixes found in an urban airshed. Also important was a realistic injection of random errors characterizing pure source profiles as well as “measured” ambient samples. This was accomplished by means of the linear equation given below, where the *S_jt_* were generated by applying a dispersion model incorporating real meteorological data to two urban (geographic) models. The STD generation scheme is illustrated for one of these urban models in figure 8.

#### Generating Equation

$${\tilde{C}}_{it}=\overset{P}{\Sigma }[\tilde{A}-e_{m}-e_{H}]{\;}_{ij}S_{jt}+e_{it},$$

where:

*t*: = sampling period [1 ≤ *t* ≤ 40]
${\tilde{C}}_{it}$: = “observed” concentration of species—*i*, period—*t* [1 ≤ *i* ≤ *N, N* ≤ 20]
*S_jt_*: = true intensity (at receptor) of source—*j* [l ≤ *j* ≤ *P, P* ≤ 13]
${\tilde{A}}_{ij}$: = “observed” source profile matrix (element—*i,j*)
*e_i_*: = random measurement errors, independent and normally distributed
e_m_: = systematic source profile errors, independent and normally distributed (systematic, because fixed over the 40 sampling periods)
*e*_H_: = random source profile variation errors, independent and log-normally distributed

The outcome of the exercise was instructive. Though results for the several techniques were generally correlated, and agreement with the “truth” was generally within a factor of two, some important differences and discrepancies were observed. For example, FA methods in contrast to weighted least squares estimation (“chemical mass balance”) could not provide estimates for all components. They were limited to a collection of four or five component classes. Also, presumably identical methods, operating on strictly identical data resulted in differing component estimates as well as different standard error (SE) estimates. Comparing the actual distributions of residuals to the quoted SEs, we found the latter to vary from gross underestimates to gross overestimates. It was clear from this exercise that results depended heavily on “operator judgment,” i.e., unique solutions could not be obtained without the use of certain, often implicit assumptions or decisions. It can be shown that problems of this sort, and in fact a large fraction of the multivariate problems in chemistry, are underdetermined or heavily dependent on assumptions. This is a challenge to chemometrics. Chemical knowledge combined with astute design should eliminate some of the inaccuracy connected with model selection, error treatment, and incautious use of criteria such as non-negativity.

Just as with SRMs, the above intercomparison was not the last word with this data set. Rather it has served as a test bed for additional and newly-developed methods of multivariate chemical data analysis [16], the most recent of which involves a new, more accurate representation of multivariate data by “parallel coordinate” systems [33]. In the future, we would expect STD to continue to serve the mutiple purposes of chemometric quality control for both conventional and automated analytical systems, assessment of interlaboratory or interalgorithmic accuracy, and as stimuli for chemometric research on complex, multicomponent systems.

## 5. Summary and Forecast

In conclusion, let us consider for a moment the matter of forecast, as viewed from two perspectives: (1) What may be forecast for the future of chemometrics in relation to standards and accuracy? (2) What directions are envisioned if we are to use chemometrics to improve our ability to understand and forecast the behavior of external systems, such as the environment? Key issues which comprise the answer to the *first question* are:

- Nomenclature, including rigorous terminology and formulation of the performance characteristics of the CMP, plus standard nomenclature for methods of CMP design, control and evaluation derived from applied mathematics.
- Optimal design of the overall analytical system to meet prescribed analytical needs and accuracy limits, utilizing detailed chemical knowledge of the characteristics of the individual CMP steps.
- Attention to the validity of the analytical model, both the functional relationship and the noise models; specification of hypotheses and tests having adequate power with respect chemically significant alternative hypotheses.
- Assessment of the accuracy of mathematical techniques as applied to chemical data, via algorithm or software evaluation, or overall data reduction evaluation using STD.
- Development of new methods of increased accuracy by iteratively linking CMP design, chemical separation, instrumental measurement and data evaluation, to reduce dependence on unverified assumptions, and to improve precision through interference reduction and application of expert knowledge.

The *second question* relates to the fact that data-based, empirical models cannot be relied upon to provide information beyond their immediate domain. That is, if we wish to be in a position to make accurate forecasts, or even accurate interpolations, for a given system, *there is no substitute for a detailed mechanistic understanding* of the properties (model) of that system. It is in this area that chemometrics, and analytical chemistry, have their greatest promise for the future. This prospect is best viewed in terms of a pair of interacting systems. The first system represents the *raison d’être* or driving force for analytical chemistry; it is the external system which depends on chemical analyses for its elucidation or control. The second system is the analytical system or CMP. Chemometrics has long recognized the linkage between these two systems, but much of the work has been based on sampling and measurements designed to establish empirical patterns, or “soft modeling” [34].

Soft modeling, which might be viewed as an outgrowth of empirical, statistical modeling, is extremely important for exploratory studies, and for providing statistical descriptions of empirical relationships in complex chemical or biological systems. In contrast, “hard global models… have great advantages both in their far-reaching predictions and their interpretation in terms of fundamental quantities.” And, unlike soft models, “the deviation between the hard model and the measured data must not be larger than the errors of measurement” (Wöld and Sjöstrom, pp. 243ff. [34]). Increased movement in chemometrics toward hard modeling is clearly attractive because of the potential for increased basic understanding and increased accuracy; it is realistic in view of the enormous advances during the last decade in sampling and measurement capabilities, and especially in computational capacity.

The transition toward more accurate representation of the external physical, chemical or biological systems which analytical chemistry must serve is outlined in table 5. To complement Wold’s basic categories, we present the “musical” classification of Douglas Hofstadter [35], and the mechanistic model categories often used to describe biological or environmental systems [36]. Hofstadter’s descriptors are apt. They convey succinctly the increasing sophistication of models (“analogies”) in an area of enormous intrinsic complexity—artificial intelligence. The flow of models for the environmental system brings us immediately back to analytical chemistry and chemometrics. That is, the linear model, such as that described in section 4.3 is our simplest representation for an environmental system. Consistency and accuracy, governed by measurement error alone, cannot be generally expected with so simple a model. Improvements may be gained through: (1) combined chemometric techniques, such factor analysis followed by time series analysis, to explore the dynamics of the system [37]; and (2) “hybrid” modeling to take into account certain non-linearities such as homogeneous and heterogeneous reactions [38]. Major progress in understanding and monitoring an environmental system comes when natural “compartments” may be defined, with differential equations describing transfers between compartments [39]. When the compartmental description is inadequate, one must consider an even more detailed description of the system, generally by taking into consideration its full dynamic space-time character through the use of coupled equations representing transport and reaction [40]. These last two categories of modeling and measurement are important for assessing the potential impact of human activities on climate, in connection with the “CO_2_” problem, and the coupled reactive system CO-OH-CTL_4_, respectively [41].

We face very important opportunities to gain increased fundamental knowledge of the nature (mechanistic models) and state of external (environmental, biological) systems through the use of hard, or at least harder, models to guide the sampling and measurement designs for these systems. By working closely with expert theoretical geochemists or biochemists, for example, chemometricians have the opportunity to design the analytical measurement process to optimally test alternative external models, to better estimate their parameters, and to more accurately evaluate their present state and future course [42].

## Figures and Tables

**Table 1:** What is chemometrics?

1.	NATO Advanced Study Institute (1983)	
	“Chemometrics: Mathematics and Statistics in Chemistry”	
2.	IUPAC—Working Group on Chemometrics (1987)	
	Scope	
	Producing Chem. Information	Notation & Terminology
	Precision & Accuracy: intralab, interlab	
	Calibration: univariate, multivariate	Relating Chemical & Non-Chemical Data
	Information Theory	Performance Characteristics
	Optimization *&* Exptl. Design: sequential, simultaneous	
	Signal Analysis: 4 chapters	Data Analysis: 8 chapters
	Expert Systems: custom made, knowledge engineering tools	
	Operations Research	Graph Theory
		Robotics
	Computational Techniques (future strategies)	
	Chemical Image Analysis	Sampling Strategies
	Quality Control	Systems Theory

**Table 2:** A brief history

IUPAC (1987):	Report on Chemometrics (D.L. Massart, M. Otto)
Two textbooks:	“Chemometrics: a textbook” (1987, Elsevier) (Massart, Vandeginste, Deming, Michotte, Kaufman)
	“Chemometrics” (1986, Wiley) (Sharaf, Illman, Kowalski)
Two Journals:	Journal of Chemometrics (Jan. 1987) (Ed. Kowalski, Wiley)
	Chemometrics and Intelligent Laboratory Systems (Nov. 1986) (Ed. Massart; Elsevier)
Chemometrics Conference:	(NBS, May 1985)—dedicated to W. J. Youden (Spiegleman, Sacks, Watters; NBS J. Research 90 [6])
NATO Advanced Study Institute on Chemometrics:	
	(Cosenza, Sept. 1983) (Kowalski)
“Chemometrics: Theory and Application” (1977)	
	(Ed. Kowalski; ACS Sympos 52)
Chemometrics Society founded (Seattle, 1974) (S. Wold, B. Kowalski)	
CONCEPTION—S. Wold (1971) (J. Chemometrics, V. 1, No. 1, p. 1, Jan. 1987)	
Analytical Chemistry (ACS),	
Reviews on statistics … mathematics… chemometrics (even years)	
W. J. Youden, “Statistical Methods for Chemists” (1951, Wiley)	

**Table 3:** Chemometric standards

Nomenclature (terminology, concepts, formulation)
Standards for accuracy (entire chemical measurement process)
detection, identification, estimation, uncertainties, assumptions
evaluation of chemometric techniques, software, algorithms
validation through “standard” data; interlaboratory exercises
design to meet external needs for adequate, accurate chemical information
Advance the state of the art; stimulate multidisciplinary cooperation

**Table 4:** Analytical accuracy and hypothesis testing^a^

Hypothesis formation (external system model)
Design of the measurement process—external (*x*, *l*, *t*) —internal (MP, EP)
Hypotheses to be tested:
model (simplest internal; *y =B +Ax +e_y_*)
detection, discrimination (estimation)
no. of components (knowledge, “fit,” constraints)
identification (informing variable; pattern)
error structure (stationary, white, cdf, variance, bias)
Some diagnostics—*z, t, t*′, K-S, χ^2^, χ^2^′, *F*, residual patterns, …

a Symbol explanation: *x, l*, *t* = sampling species, location(s), time(s), MP, EP = measurement and evaluation steps of the CMP, *t*′, χ^2^′ = noncentral *t* and χ^2^ statistics; K-S = Kolmogorov-Smirnov statistic.

**Table 5:** The transition from empirical to mechanistic modeling

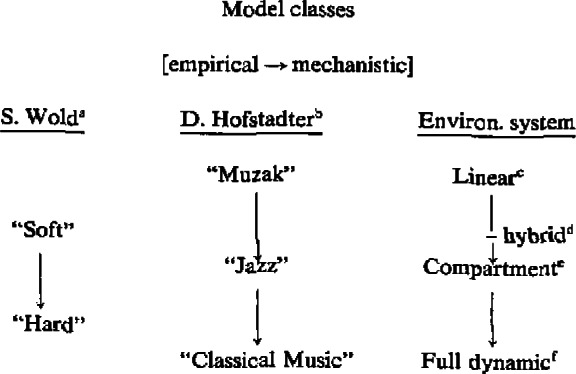

a See reference [34].

b See reference [35].

c Multivariate source apportionment (conservative tracers) [32].

d Particle—sulfate system apportionment [37,38].

e CO_2_ system: troposphere-biosphere-ocean; biological systems [36,39].

f CO-OH-CH_4_ system (production, transport, reaction) [40,41].
